# Resveratrol Inhibits Ionising Irradiation-Induced Inflammation in MSCs by Activating SIRT1 and Limiting NLRP-3 Inflammasome Activation

**DOI:** 10.3390/ijms140714105

**Published:** 2013-07-08

**Authors:** Yue Fu, Yan Wang, Liqing Du, Chang Xu, Jia Cao, Tiqiang Fan, Jianxiang Liu, Xu Su, Saijun Fan, Qiang Liu, Feiyue Fan

**Affiliations:** 1 Tianjin Key Lab of Molecular Nuclear Medicine, Institute of Radiation Medicine of Chinese Academy of Medical Science and Peking Union Medical College, Tianjin 300192, China; E-Mails: wddr0710@sina.com (Y.F.); bamboo201306@163.com (Y.W.); duliqing2004@126.com (L.D.); xuchang2001@yahoo.com (C.X.); jillpumc@gmail.com (J.C.); fantiqiang2008@sina.com (T.F.); fansaijun@gmail.com (S.F.); 2 National Institute for Medical Radiological Protection, Chinese Center for Disease Control and Prevention, Beijing 100088, China; E-Mails: jxliu@nirp.cn (J.L.); suxu@nirp.cn (X.S.)

**Keywords:** radiation, IL-1β, SIRT1, NLRP3, resveratrol

## Abstract

IL-1β, a pro-inflammatory cytokine, has been shown to contribute to radiation injury. Sirt1, an NAD^+^-dependent class III protein deacetylase, plays an important role in the regulation of the proinflammatory cytokines involved in inflammation-associated diseases. The relationship between Sirt1 and IL-1β, however, has remained elusive. The present study was designed to explore the potential effect of Sirt1 on IL-1β expression induced by radiation and to provide a new target for the development of radiation protection drugs. Our results showed that radiation significantly increased IL-1β mRNA and protein expression and that pretreatment with resveratrol, a Sirt1 activator, inhibited the radiation-induced IL-1β expression in a concentration-dependent manner, whereas the knockdown or inhibition of Sirt1 by nicotinamide significantly enhanced radiation-induced IL-1β expression. This effect can likely be attributed to Sirt1-mediated inhibition of NLRP-3 inflammasome activation because Sirt1 inhibits the transactivation potential of NF-κb by deacetylation, which then suppresses NLRP3 transcription. Taken together, the results demonstrate that Sirt1 exerts anti-inflammatory effects by regulating NLRP3 expression partially through the NF-κb pathway in mesenchymal stem cells. More importantly, our findings suggest that resveratrol is an effective agent in protecting against radiation injury, and we provide a theoretical basis for developing a drug to protect against radiation injury by targeting Sirt1.

## 1. Introduction

Mesenchymal stem cells (MSCs) are multipotent cells that can be isolated from several human tissues and expanded *ex vivo* for clinical use [1,2]. MSCs show significant potential for clinical utility due to their convenient isolation and culture conditions, low immunogenicity, regenerative and differentiation abilities, and potent immunosuppressive effects [3]. Because of these properties, an increasing number of studies suggest that the role of MSCs needs to be explored in the clinical treatment of severe radiation injuries such as radiation-induced lung injury and post-irradiation salivary gland damage [4–6]. However, MSCs are more sensitive to radiation, and we thus focused on investigating the mechanism of radiation damage in mesenchymal stromal cells to explore drugs that protect stems cells from radiation damage.

Sirtuin 1 (Sirt1), the mammalian Sir2 homologue, is a class III histone deacetylase shown to act on a wide range of histones and non-histone substrates including NF-κb, p53, and PGC-1α [7,8]. Sirt1 can mediate a variety of physiological events, including cell survival, metabolic rate, and oxygen consumption, via the deacetylation of target substrates [9]. Recent studies have shown that Sirt1 interferes with the NF-κb signalling pathway and therefore has an anti-inflammatory function. For example, Sirt1 can interact with the p65 subunit of NF-κb and inhibit transcription by deacetylating p65 at Lys310 and then suppressing the inflammatory factor [10,11]. This interaction indicates that the anti-inflammatory and cell-protective effects of Sirt1 may prove useful in treating radiation injury.

The NLRP3 inflammasome is currently the most fully characterised inflammasome and consists of the NLRP3 scaffold, the ASC (PYCARD) adaptor, and caspase-1. Pathogen- and damage-associated molecular pattern molecules and environmental irritants can activate NLRP3 [12]. Once the NLRP3 inflammasome is activated, it is able to convert inactive pro-IL-1β into its bioactive and secreted forms [13]. The NLRP3 inflammasome assembles in response to a variety of diverse exogenous and endogenous activators, including various microbial signals (bacteria, fungi, and viruses), pore-forming toxins, crystalline substances, peptide aggregates, and extracellular ATP that is released from dying cells [14–16]. The mechanism by which the NLRP3 inflammasome is activated is unknown; all that is known is that it is required for NF-κB activation, which is the traditional priming signal, and induces the transcription of NLRP3 and pro-IL-1β [17]. Functionally, ROS were proposed to be exclusively involved in NLRP3 activation because they can promote the dissociation of thioredoxin-interacting protein (TXNIP) from thioredoxin (TRX), which allows it to directly bind to and activate NLRP3 [18]. As is known, ionising radiation can increase ROS levels and IL-1β expression. IL-1β is a pro-inflammatory cytokine that is the most important of all cytokines due to its central role in the inflammatory process, but the mechanism by which the expression of IL-1β is increased due to radiation is unknown.

Resveratrol (3,4,5-trihydroxy-trans-stilbene) is a natural non-flavonoid polyphenolic found in the skin of red grapes [19]. Many studies have shown that resveratrol can prevent or slow the progression of a variety of conditions, including cancers, cardiovascular diseases, and ischemic injuries, and can enhance stress resistance and extend lifespan [20,21]. As a polyphenolic compound, resveratrol is frequently used as an activator of Sirt1; it has also been shown to be a scavenger of hydroxyl, superoxide, and metal-induced radicals [22,23]. Recently, mice given resveratrol before radiation were shown to have increased survival rates [24]. It is unknown whether resveratrol activates Sirt1 to suppress inflammation induced by radiation, and it is also unknown which intracellular signalling pathways contribute to this phenomenon.

In our study, because Sirt1 inhibits NF-κb transcriptional activity through deacetylation and NLRP3 transcription requires NF-κb, we propose that resveratrol exerts anti-inflammatory effects by activating Sirt1 and limiting NLRP3 transcription.

## 2. Results and Discussion

### 2.1. Radiation Elevates IL-1β Levels in MSCs after Radiation

**Figure 1:**
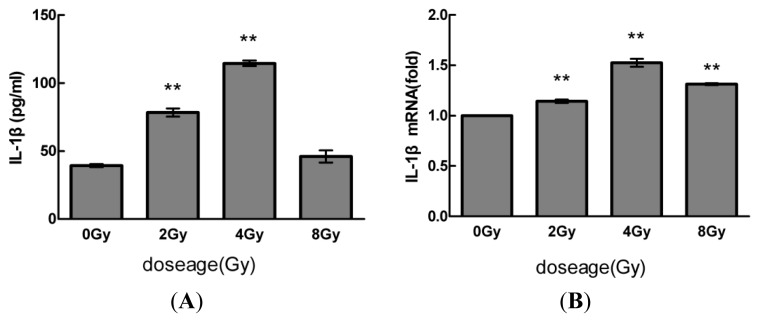

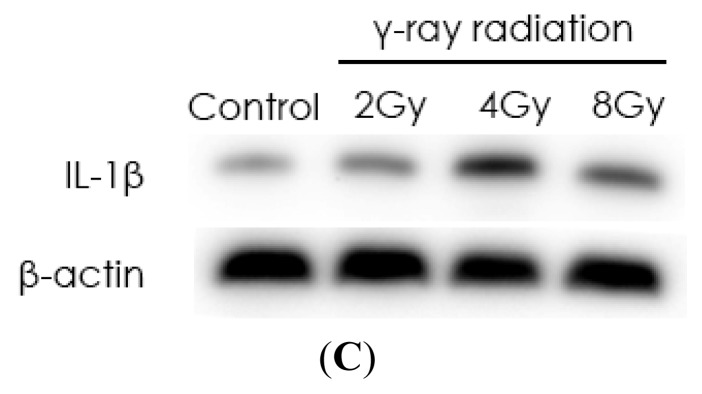
Radiation elevates IL-1β levels in MSCs after radiation. (**A**) Dose-dependent increase in IL-1β secretion and (**B**,**C**) dose-dependent increases in IL-1β mRNA and protein expression. Cells were exposed to various doses of radiation (0, 2, 4, and 8 Gy), and after 24 h, extracellular and intracellular protein and mRNA levels of IL-1β were determined by ELISA, Western blot, and quantitative real-time PCR analyses, respectively. The values are presented as the mean ± SD (*n* = 3). *p < 0.05, **p < 0.01 compared with the control (0 Gy) group.

We first measured IL-1β secretion levels in cell culture supernatants by ELISAs after exposure to various doses of radiation (0, 2, 4, and 8 Gy). As shown in Figure 1A, IL-1β levels in supernatants were elevated 24 h after radiation. IL-1β supernatant levels reached a maximum in cells exposed to 4 Gy. We then detected IL-1β levels from MSC cell lines by Western blot and RT-PCR analyses. As shown in Figure 1B,C, consistent with the ELISA results, radiation caused a dose-dependent increase in IL-1β mRNA and protein levels.

### 2.2. Resveratrol Reduces IL-1β Expression in MSCs

**Figure 2:**
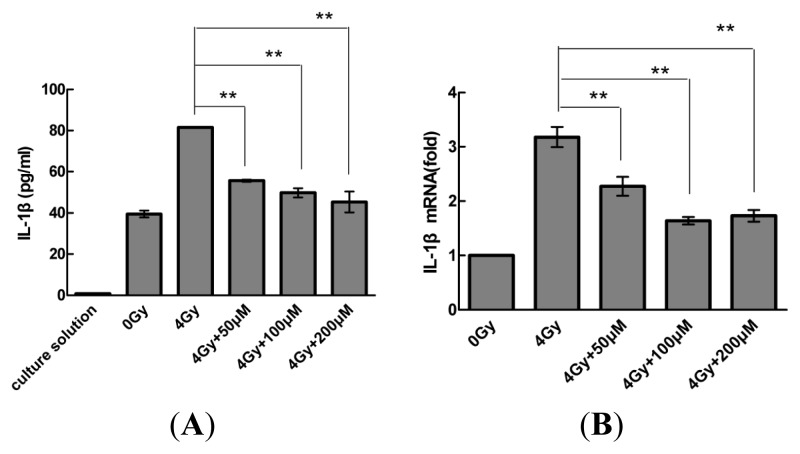

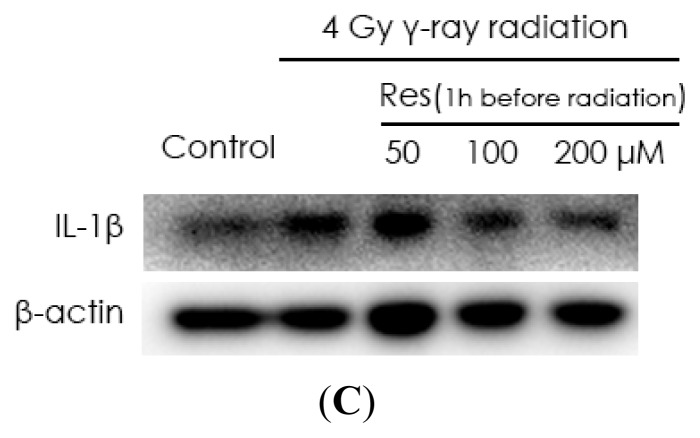
Resveratrol reduces IL-1β expression in MSCs. (**A**) Concentration-dependent decrease in IL-1β secretion and (**B**,**C**) concentration-dependent decrease in IL-1β protein and mRNA expression. Cells were treated with different concentrations of resveratrol for 1 h before radiation. Then, extracellular and intracellular protein and mRNA levels of IL-1β were determined by ELISA, Western blot, and quantitative real-time PCR analyses, respectively. Values are presented as the mean ± SD (*n* = 3). *p < 0.05, **p < 0.01 compared with the radiation group.

To investigate the effect of resveratrol on IL-1β, MSCs were pre-treated with resveratrol (0, 50, 100, or 200 μM) for 1 h before radiation. At 24 h after being exposed to 4-Gy radiation, protein and mRNA levels of IL-1β were determined by Western blot and quantitative real-time PCR analyses, respectively; IL-1β secretion levels were assayed by performing ELISAs on cell culture supernatants. As shown in Figure 2A–C, resveratrol caused a concentration-dependent decrease in IL-1β expression to a similar level as that observed in control cells. The ELISA results also showed that extracellular levels of IL-1β decreased in a concentration-dependent manner.

### 2.3. Sirt1 Is Upregulated in Resveratrol-Treated Cells

**Figure 3:**
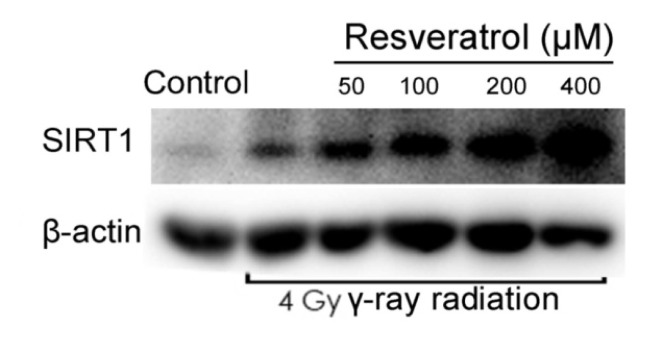
Sirt1 is upregulated in MSCs treated with resveratrol. MSCs were treated with the indicated concentrations of resveratrol, and the relative expression of Sirt1 was determined by Western blot analysis. Values are presented as the mean ± SD (*n* = 3). *p < 0.05, **p < 0.01 compared with controls.

Protein expression of Sirt1 was upregulated by resveratrol (50, 100, 200, or 400 μM) in a concentration-dependent manner. Although the Sirt1 levels were highest at doses of 400 μM, this concentration was also toxic (data not shown). Regardless, non-toxic doses of 200 μM resveratrol still significantly increased Sirt1 levels (Figure 3).

### 2.4. Pharmacological Modulation of Sirt1 Regulates Radiation-Induced NLRP3 and IL-1β Expression in MSCs

**Figure 4:**
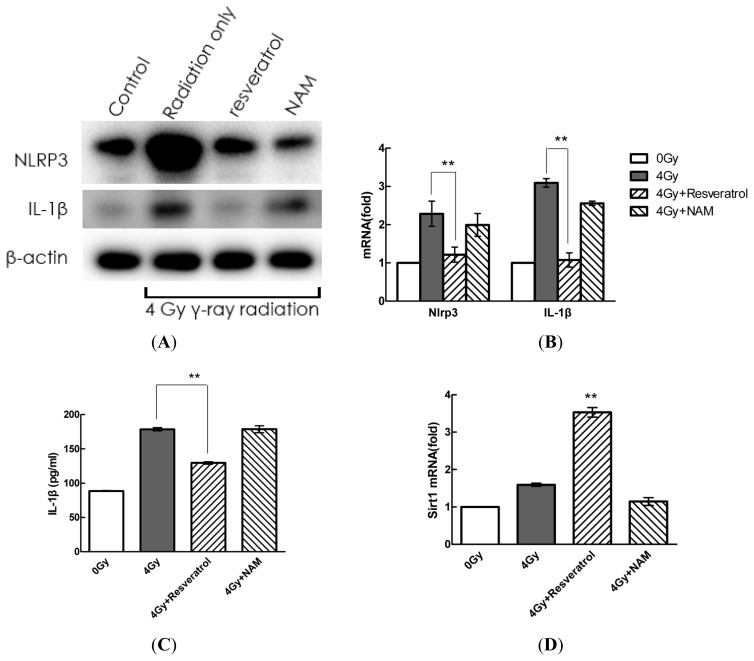
Pharmacological modulation of Sirt1-regulated radiation-induced NLRP3 and IL-1β expression in MSCs. (**A**–**D**) Cells were pre-treated with resveratrol (200 μM) or NAM (20 mM) and subsequently stimulated with radiation for 24 h. Then, extracellular and intracellular protein and mRNA expression of IL-1β were determined by ELISA, Western blot, and quantitative real-time PCR analyses, respectively. The values are presented as the mean ± SD (*n* = 3). *p < 0.05, **p < 0.01 compared with the radiation group.

To assess the association between Sirt1 and IL-1β expression, MSCs were pre-treated with the Sirt1 activator resveratrol (200 μM) and Sirt1 inhibitor NAM (20 mM) for 1 h before radiation exposure. Twenty-four hours after radiation exposure, the protein and mRNA levels of IL-1β and NLRP3 were determined by Western blot and quantitative real-time PCR analyses, respectively, and IL-1β secretion levels were detected by ELISAs. As shown in Figure 4A–C, the pre-treatment of MSCs with resveratrol attenuated IL-1β and NLRP3 expression induced by radiation. However, pre-treatment with NAM decreased Sirt1 expression (Figure 4D) and significantly augmented IL-1β and NLRP3 expression.

### 2.5. Knockdown of Sirt1 by shRNA Suppresses Resveratrol-Mediated Anti-Inflammatory Activity

**Figure 5:**
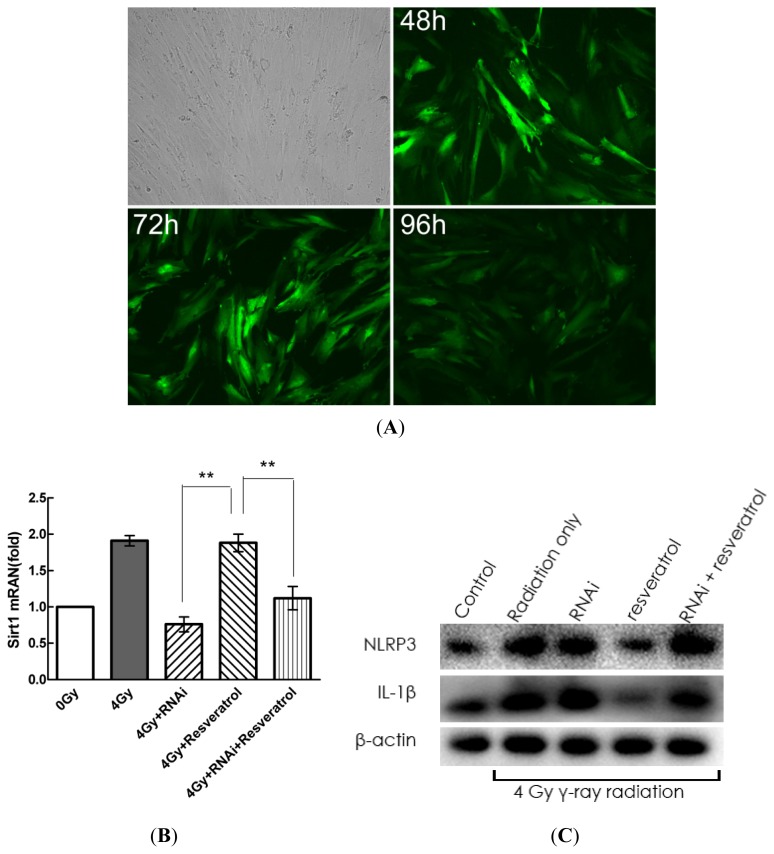

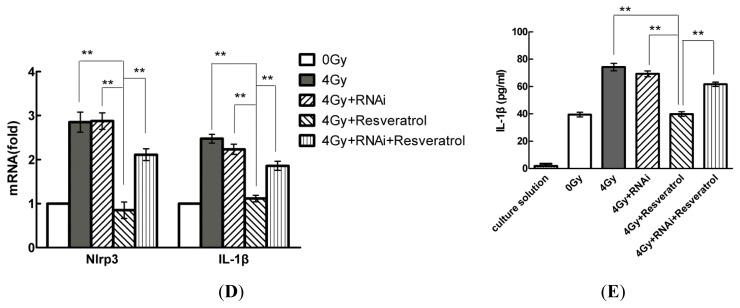
The knockdown of Sirt1 by shRNA suppresses resveratrol-mediated anti-inflammatory activity. (**A**) Cells were transfected with Sirt1 shRNA for 48, 72, or 96 h; (**B**) Sirt1 expression was down-regulated by Sirt1 shRNA. The cells were transfected with Sirt1 shRNA for 48 h, and mRNA expression of Sirt1 was then determined by quantitative real-time PCR; (**C**–**E**) The knockdown of Sirt1 obviously suppresses resveratrol-mediated anti-inflammatory activity, and radiation-induced IL-1β expression was significantly increased by Sirt1 RNAi. The cells were stimulated with radiation for 24 h after transfection with Sirt1 shRNA for 48 h; protein and mRNA expressions of IL-1β and NLRP3 were determined by Western blot and quantitative real-time PCR analyses, respectively, and IL-1β secretion was detected by ELISAs. Values are presented as the mean ± SD (*n* = 3). *p < 0.05, **p < 0.01 compared with the resveratrol group.

To further confirm the role of Sirt1 in radiation-induced inflammation in MSCs, cells were transfected with Sirt1 shRNA for 48, 72, and 96 h. As shown in Figure 5A, after 48 h, robust GFP expression was observed in MSCs, and GFP expression was still observed after 72 and 96 h. The mRNA expression of Sirt1 was determined using real-time PCR. As expected, the cells transfected with Sirt1 shRNA exhibited a lower expression of Sirt1 mRNA (Figure 5B). Then, Sirt1 knockdown cells were pre-treated with resveratrol (200 μM) for 1 h before radiation exposure. At 24 h after radiation exposure, the intracellular protein and mRNA expression of IL-1β and NLRP3 were determined by Western blot and quantitative real-time PCR analyses, respectively, and IL-1β secretion was detected by ELISAs. As shown in Figure 5C–E, compared with the resveratrol group, the knockdown of Sirt1 obviously suppresses resveratrol-mediated anti-inflammatory activity, and the expression and secretion of IL-1β were much higher.

### 2.6. Sirt1 Inhibits IL-1β Expression via the NF-κb Pathway in MSCs

**Figure 6:**
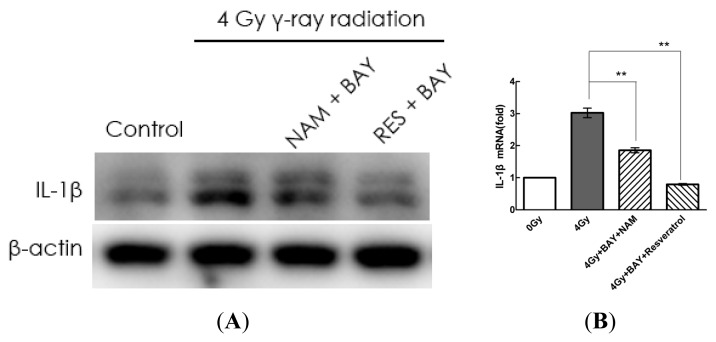

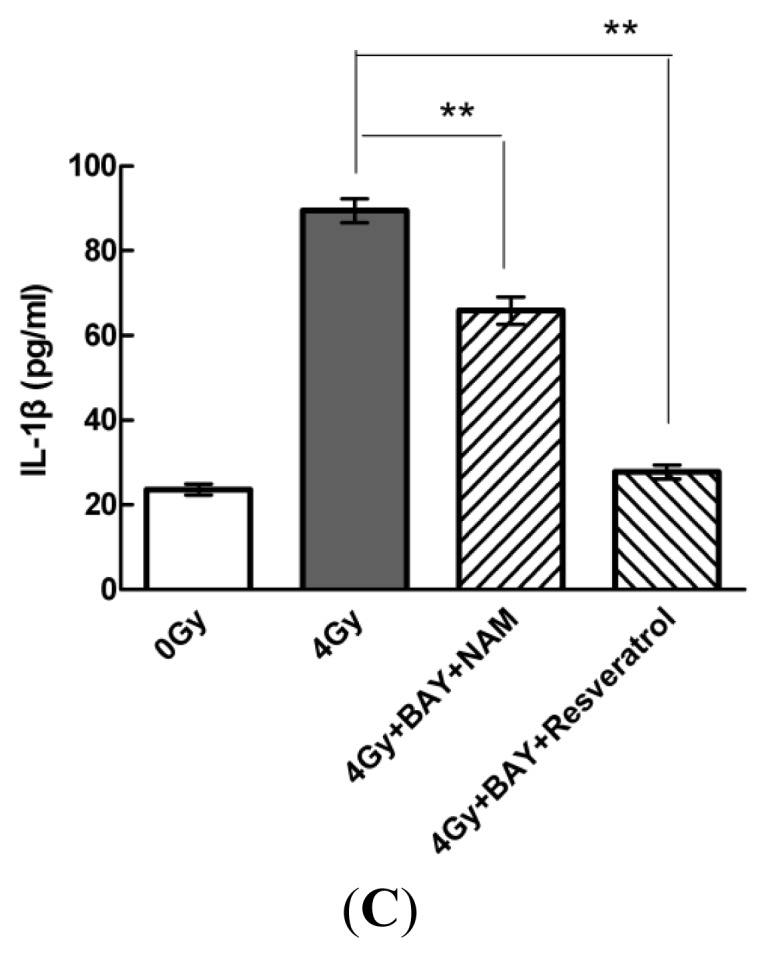
Sirt1 inhibits IL-1β expression via the NF-κb pathway in MSCs. (**A**–**C**) Cells were pre-treated with BAY (5 μM) and either resveratrol (200 μM) or NAM (20 mM) for 1 h prior to stimulation with radiation (4 Gy). Extracellular and intracellular protein and mRNA levels of IL-1β were determined by ELISA, Western blot, and quantitative real-time PCR analyses, respectively. Values are presented as the mean ± SD (*n* = 3). *p < 0.05, **p < 0.01 compared with the radiation group.

To investigate whether the NF-κb pathway was associated with radiation-induced IL-1β expression, we applied BAY, an inhibitor of NF-κb, and observed its effects on IL-1β expression associated with Sirt1 activation or inhibition. MSCs were treated with BAY (5 μM) and either resveratrol (200 μM) or NAM (20 mM) for 1 h and subsequently stimulated with radiation. We found that compared with the radiation group, resveratrol and BAY inhibited the expression of IL-1β induced by radiation. The inhibitory effect of the combined treatment with resveratrol and BAY was higher than that of the combination treatment with BAY and NAM (Figure 6A–C).

## 3. Discussion

IL-1β is a very important inflammatory factor. After radiation therapy, the expression of IL-1β increases and leads to cell apoptosis and tissue damage. MSCs show significant potential for clinical utility due to their low immunogenicity, regenerative and differentiation abilities, and potent immunosuppressive effects [3]. Studies also suggest that MSCs can be applied to the treatment of radiation injury. However, because MSCs are more sensitive to radiation, we used *in vitro* MSCs to explore the role of Sirt1 in radiation-induced inflammation; we then provided a theoretical basis for developing a radioprotective drug that targets Sirt1.

Resveratrol modulates the synthesis of lipids, lipid catabolism, and apoptosis, and it possesses anti-cancer and anti-inflammatory properties [25]. Zhang et al. reported that mice given resveratrol before radiation had significantly higher survival rates, which is due, at least in part, to resveratrol’s regulation of superoxide dismutase and glutathione peroxidase [23]. Moreover, Şimşek and Simsek also reported that resveratrol could ameliorate salivary gland and ovarian damage induced by radiation [26,27]. Most research to date has focused on the antioxidant properties of resveratrol, rather than on the effect of resveratrol suppression of inflammation. In this study, we found that IL-1β was expressed in MSCs after radiation and that its secretion levels were also increased in a dose-dependent manner. Pre-treating MSCs with resveratrol for 1 h before radiation led to a suppression of IL-1β secretion, which was induced by radiation in a dose-dependent manner. Meanwhile, we observed that as the concentration of resveratrol increased, the protein and mRNA expression of IL-1β were significantly suppressed. This result suggests that resveratrol may inhibit the secretion of IL-1β by down-regulating the protein and mRNA levels of IL-1β.

Because resveratrol has been known as a Sirt1 activator [28], to further understand the anti-inflammatory mechanism of resveratrol in MSCs, we investigated whether Sirt1 played a key role in resveratrol’s protective effect. Sirt1 is an NAD^+^-dependent deacetylase that regulates lipid and glucose homeostasis and is the proinflammatory cytokine involved in inflammation-associated diseases [29]; in fact, the activation of Sirt1 has been shown to inhibit TNF-α-induced inflammation in fibroblasts [30]. Our data show that resveratrol significantly elevates the expression of Sirt1. Compared with the resveratrol group, both RNAi of Sirt1 and treatment with NAM, a Sirt1 inhibitor, suppressed resveratrol-mediated anti-inflammation, indicating that resveratrol inhibits IL-1β expression induced by radiation in a Sirt1-dependent manner.

Several reports have suggested that IL-1β is initially produced as a pro-peptide and is processed into its mature cytokine by caspase-1, which occurs in a multi-protein complex with NLRP3 and ASC, termed the inflammasome [31]. In our study, we found that radiation-induced IL-1β expression is associated with NLRP3. At high expression levels of NLRP3, the secretion and expression of IL-1β were also significantly increased. This result demonstrates that radiation-induced increases in IL-1β occur via the NLRP3 pathway.

NF-κb is a transcription factor that is essential for the development of most inflammatory responses [32]. The activation of NF-κb increases the transcription of the IL-1β gene encoding pro-IL1β and thus elevates the intracellular levels of the procytokine; furthermore, NLRP3 transcription is also dependent on NF-κb [17]. Because NF-κb is essential for the secretion of IL-1β induced by radiation and because resveratrol is a Sirt1 activator, we hypothesised that NF-κb and Sirt1 may functionally interact in MSCs. Our results suggest that combined with either resveratrol or NAM, BAY can obviously inhibit IL-1β secretion and expression. More importantly, NF-κb was regulated by post-translational modifications such as the reversible acetylation of p65, which thereby down-regulates the expression of various pro-inflammatory cytokines [33,34]. We hypothesised that the functional interaction between Sirt1 and p65 may involve the deacetylation of Lys310 in p65, which then leads to the suppression of NF-κb activity.

## 4. Experimental Section

### 4.1. Cell Lines

Human umbilical cord blood-derived mesenchymal stem cells were a gift from the National Engineering Research Centre of Human Stem Cells. Cells were grown in 25-cm^2^ flasks containing 3 mL of 1X DMEM/F12 (Hyclone, UT, USA) supplemented with 10% foetal bovine serum (FBS) and 1% penicillin/streptomycin in a humidified incubator containing 5% CO_2_ at 37 °C.

### 4.2. Reagents and Ionising Radiation

Resveratrol and BAY11-7082 were purchased from Sigma (St. Louis, MO, USA). Resveratrol was prepared as a 50 mM solution in DMSO, while BAY11-7082 was prepared as a 20 mM solution in DMSO. NAM was purchased from Beyotime Institute of Biotechnology (Haimen, Jiangsu, China) and was prepared as a 1 M solution in PBS. Rabbit polyclonal CIAS1/NALP3, rabbit polyclonal IL-1β, and mouse monoclonal β-actin antibodies (mAbcam 8226) were obtained from Abcam. Rabbit polyclonal SIRT1 was purchased from Santa Cruz Biotechnology (Shanghai, China).

Cells were exposed to ionising radiation (IR) in a Cammacell-40 ^137^Cesium γ irradiator (Atomic Energy of Canadian Inc., Mississauga, Canada) at a rate of 0.71116 Gy/min.

### 4.3. Vector Construction of Sirt1 shRNA and Lentiviral Infection

The expression vector of Sirt1 shRNA was constructed with the lentiviral plasmid pGCSIL-GFP (GeneChem, Shanghai, China). Age and EcoR sticky ends were included in the 5′ end of the upstream and downstream primers, respectively. Scrambled shRNA oligonucleotides (sense, 5′-CCGGGCGGGAATCCAAAGGATAATTCTCGAGAATTATCCTTTGGATTCCCGCTTTTTG-3′; antisense, 5′-AATTCAAAAAGCGGGAATCCAAAGGATAATTCTCGAGAATTATCCTTTGGATTCCCGC-3′) were annealed and cloned into pGCSIL-GFP at Age and EcoR sites.

LV-shSirt1, the pHelper 1.0 vector, and the VSV-Genvelope protein plasmid were co-transfected into 293T packaging cells according to the manufacturer’s instructions. After transfection (48 h), the supernatant containing viral particles was collected and passed through a 0.45-μM filter to remove cellular debris. MSCs were plated at 2 × 10^5^ cells per well and cultured in DMEM/F12 medium containing 10% FBS. Upon reaching 70%–80% confluence, the viral suspension of LV-Sirt1 shRNA was added. After infection (48 h), cells were treated with resveratrol and radiation and were harvested for ELISA, RT-PCR, and Western blot analyses.

### 4.4. Enzyme-Linked Immunosorbent Assay (ELISA)

IL-1β secretion was evaluated from cell suspensions collected 24 h after radiation exposure. The IL-1β ELISA was performed according to the manufacturer’s instructions (R& D Systems, Minneapolis, MN, USA). All assays were performed in triplicate. Protein levels were calculated as pg/mg of total protein.

### 4.5. RNA Extraction, cDNA Synthesis, and Quantitative Real-Time PCR

Total RNA was prepared using Trizol (Invitrogen, Inc., Carlsbad, CA, USA) according to the manufacturer’s instructions. Equal amounts of total RNA were reverse-transcribed using a PrimeScript RT reagent Kit (TaKaRa, Dalian, China) per the manufacturer’s recommendations. Quantitative real-time PCR (qRT-PCR) was performed on an ABI Prism 7500 Sequence Detection System (Applied Biosystems Inc.) with Fast SYBR Green Master Mix and a StepOnePlus instrument (Roche) according to the manufacturer’s protocol. Triplicate samples were analysed. PCR primers for the NLRP3, IL-1β, caspase-1, Sirt1, and GAPDH genes were obtained from Sangon Biotech (Shanghai, China). The specific primer pairs used were as follows: Sirt1, 5′-GACTTCAGGTCAAGGGAT-3′ (forward) and 5′-CGTGTCTATGTTCTGGGTA-3′ (reverse); NLRP3, 5′-ACAGCATTGAAGAGGAGTGGA-3′ (forward) and 5′-TCGTGTGTAGCGTTTGTTGAG-3′ (reverse); IL-1β, 5′-GTGGCAATGAGGATGACTTGT-3′ (forward) and 5′-TGTAGTGGTGGTCGGAGATTC-3′ (reverse); caspase-1, 5′-ATGCCCACCACTGAAAGAGT-3′ (forward) and 5′-ACTTCCTGCCCACAGACATT-3′ (reverse); GAPDH, 5′-ATGACATCAAGAAGGTGGTG-3′ (forward) and 5′-CATACCAGGAAATGAGCTTG-3′ (reverse).

### 4.6. Western Blot Analysis

Cells were seeded in 25-cm^2^ culture flasks. Cells were washed twice with PBS and lysed at 10^4^ cell/μL in lysis buffer (M-PER Mammalian Protein Extraction Reagent, Thermo Inc.) on ice for 10 min. After the removal of cell debris by centrifugation at 14,000 rpm, the protein concentration in cell lysates was determined using the Bradford assay. Samples containing equal amounts of protein were mixed with loading buffer with 5% 2-mercaptoethanol, heated for 5 min at 95 °C, loaded onto a 10% SDS-PAGE gel, and transferred to polyvinylidene difluoride membranes (Millipore, MA, USA). After blocking with 5% milk and 0.1% Tween-20 in Tris-buffered saline (TBS), membranes were incubated overnight at 4 °C with a primary antibody against Sirt1 (1:200), NLRP3 (1:2,000), IL-1β (1:500), or β-actin (1:5000). The membranes were then incubated with the appropriate horseradish peroxide-conjugated secondary antibody for 2 h at room temperature. The target proteins were detected with an enhanced chemiluminescent detection system according to the manufacturer’s protocol.

### 4.7. Statistical Analysis

Data were expressed as the mean ± SEM. Data were subjected to statistical analysis by one-way analysis of variance (ANOVA), followed by an LSD test. *p* < 0.05 was considered statistically significant.

## 5. Conclusions

In conclusion, our results demonstrate that resveratrol inhibits IL-1β expression induced by radiation via the activation of Sirt1. Because Sirt1 activation inhibits the transactivation activity of NF-κb, which suppresses NLRP3 transcription and subsequent IL-1β production, we conclude that Sirt1 can effectively regulate the NLRP3 inflammasome. Further studies should use a radiation-induced enteritis mouse model to confirm the underlying mechanisms by which Sirt1 protects against radiation-induced inflammation. Given that resveratrol is an activator of Sirt1, our findings suggest that resveratrol is an effective protection agent for radiation-induced injury. We also provided a theoretical basis for developing radioprotective drugs that target Sirt1.
